# A Functional Polymorphism of the MAOA Gene Modulates Spontaneous Brain Activity in Pons

**DOI:** 10.1155/2014/243280

**Published:** 2014-05-25

**Authors:** Hui Lei, Xiaocui Zhang, Xin Di, Hengyi Rao, Qingsen Ming, Jibiao Zhang, Xiao Guo, Yali Jiang, Yidian Gao, Jinyao Yi, Xiongzhao Zhu, Shuqiao Yao

**Affiliations:** ^1^The Medical Psychological Institute of the Second Xiangya Hospital, Central South University, Changsha, Hunan 410011, China; ^2^Department of Biomedical Engineering, New Jersey Institute of Technology, Newark, NJ 07102, USA; ^3^Center for Functional Neuroimaging, Department of Neurology, University of Pennsylvania, Philadelphia, PA 19104, USA; ^4^Department of Psychology, Sun Yat-Sen University, Guangzhou, Guangdong 510275, China

## Abstract

*Objective*. To investigate the effects of a functional polymorphism of the monoamine oxidase A (MAOA) gene on spontaneous brain activity in healthy male adolescents. *Methods*. Thirty-one healthy male adolescents with the low-activity MAOA genotype (MAOA-L) and 25 healthy male adolescents with the high-activity MAOA genotype (MAOA-H) completed the 11-item Barratt Impulsiveness Scale (BIS-11) questionnaire and were subjected to resting-state functional magnetic resonance imaging (rs-fMRI) scans. The amplitude of low-frequency fluctuation (ALFF) of the blood oxygen level-dependent (BOLD) signal was calculated using REST software. ALFF data were related to BIS scores and compared between genotype groups. *Results*. Compared with the MAOA-H group, the MAOA-L group showed significantly lower ALFFs in the pons. There was a significant correlation between the BIS scores and the ALFF values in the pons for MAOA-L group, but not for the MAOA-H group. Further regression analysis showed a significant genotype by ALFF values interaction effect on BIS scores. *Conclusions*. Lower spontaneous brain activity in the pons of the MAOA-L male adolescents may provide a neural mechanism by which boys with the MAOA-L genotype confers risk for impulsivity and aggression.

## 1. Introduction

Monoamine oxidase (MAO) is a mitochondrial enzyme that was involved in degradation of neurotransmitters, including norepinephrine (NE), serotonin (5-HT), and dopamine [1]. There are two monoamine oxidase isozymes with distinct substrate specificities: MAOA and MAOB. MAOA provides the major enzymatic clearance of 5-HT and NE during brain development [1]. The MAOA-encoding gene (Xp11.4-Xp11.3) presents a well-characterized variable number tandem repeat (VNTR) functional polymorphism in the promoter region, with different length variants that influence protein transcription, and hence enzymatic activity, selectively [2, 3]. Enzyme expression is relatively higher in carriers of 3.5 or 4 repeats (MAOA-H allele) and lower in carriers of 2, 3, or 5 repeats (MAOA-L allele) [2]. Converging evidence indicates that this functional polymorphism has a strong influence on serotonergic function* in vitro* and* in vivo* [2–4], with the high activity allele showing lower serotonergic responsivity [4].

Previous behavioral researches have indicated an important role for MAOA in human behavior and physiology. Caspi and colleagues found that males carrying MAOA-L alleles who experienced early-life adversity had a heightened risk of developing conduct disorder or antisocial personality and of exhibiting violent and antisocial behavior [5]. Besides, Samochowiec et al. found that the MAOA-L allele has been associated with antisocial behavior in male alcohol-dependent patients [6]. Huang et al. found a significant correlation of the MAOA-H allele with lower impulsivity in adult males who report early childhood abuse, further supported an association of MAOA-L allele and impulsivity in males [7]. Subsequent studies, including one meta-analysis [8], have replicated the findings independently [9–11]. Conversely, the MAOA-H allele has been associated with an even greater propensity for antisocial behavior and impulsivity than the MAOA-L allele in males who experience early stress [12, 13]. Moreover, the MAOA-H allele has been associated with impulsive personality traits in normal male subjects [4]. Animal studies indicated that MAOA had an important effect on aggressive behavior. Aggression is increased in the male MAOA knockout mice [14] and monkeys with reared experience [15]. MAOA inhibition during brain development induced pathological aggression in mice [16].

These findings suggest that genetically driven variations in MAOA activity significantly influence impulsivity and aggression.

Using noninvasive neuroimaging techniques, including both positron emission tomography (PET) and functional magnetic resonance imaging (fMRI), previous studies have examined the effect of this polymorphism on brain function. Previous fMRI studies have demonstrated that individuals carrying MAOA-H alleles showed increased cingulate activation during conflict resolution [17] and increased orbitofrontal cortex activation during performing motor inhibition and working memory tasks [18, 19]. Meyer-Lindenberg et al. also reported that the MAOA polymorphism had a profound impact on the corticolimbic circuitry involved in emotional memory [20]. Structural MRI studies employing voxel-based morphometry (VBM) also produced conflicting findings with respect to the effect of MAOA genotypes on amygdala volume [20–22]. However, a positron emission tomography (PET) study found no differences in the glucose metabolism between MAOA-L and MAOA-H groups [23]. Currently, the neurobiological mechanisms underlying the effects of the* MAOA* polymorphism on impulsiveness are still unclear.

During the last decade, studies of spontaneous brain activity at resting states in normal individuals as well as patients with brain diseases have attracted enormous research interests. Biswal and colleagues firstly demonstrated in human subjects that spontaneous low-frequency (0.01~0.08 Hz) brain fluctuations measured by resting-state fMRI are physiologically meaningful [24]. The amplitude of low-frequency fluctuation (ALFF) of BOLD signals was developed by Zang and colleagues to provide another measurement of regional neural function during a resting-state [25]. Previous studies have suggested that ALFF is physiologically meaningful and reflective of regional spontaneous neuronal activity [25–27]. To date, ALFF analysis has been applied widely to the studies of different brain disorders, including epilepsy [28], schizophrenia [29, 30], major depressive disorder [31, 32], drug addiction [33], posttraumatic stress disorder [34], attention deficit hyperactivity disorder [25], and multiple sclerosis [35]. However, it remains unknown whether resting-state spontaneous brain activity would differ as a function of* MAOA* genetic variation. The purpose of this study is answering this question by comparing the ALFF in matched MAOA-L and MAOA-H male adolescents. Furthermore, ALFF measurements were correlated with impulsivity scores as measured by the Barratt Impulsiveness Scale (BIS) in both MAOA-L and MAOA-H genotypes, in order to examine the neural bases underlying the effects of the* MAOA* polymorphism on impulsive behavior.

## 2. Materials and Methods

### 2.1. Participants

A total of 60 healthy male adolescents were recruited from a local middle school. All of them were Han Chinese. Only male adolescents were included because the* MAOA* VNTR polymorphism maps to an X-chromosome region suspected to escape the normal X-chromosome inactivation in females [36], making homozygous females not comparable to hemizygous males in terms of enzymatic activity. The inclusion criteria were an ability to give voluntary informed consent, an absence of concurrent neurological or psychiatric disorders, no history of head trauma, alcohol or drug abuse, no history of psychiatric illness or substance abuse on the basis of a SCID I assessment for DSM-IV criteria (1994), and no history of medical treatments relevant to cerebral blood flow and metabolism. The study was approved by the Ethics Committee of the Second Xiangya Hospital at Central South University in China, and all participants gave written informed consent to participate.

### 2.2. Neuropsychological Evaluation

The Italian version of the 11-item Barratt's Impulsivity Scale (BIS-11) was administered to obtain neuropsychological profiles of the subjects' impulsivity [37].

### 2.3. Genotyping

Samples of DNA were obtained by cheek swabbing. Polymerase chain reactions (PCRs) were performed in 25 *μ*L reaction volumes containing 1 *μ*L of DNA, GoTaq Green Master Mix (Promega Company, USA), 12.5 *μ*L of each of two primers (200 ng/L), and 9.5 *μ*L ddH_2_O. The amplification protocol was as follows: 94°C for 3 min, 35 cycles of 95°C for 30 s, 58°C for 30 s, and 72°C for 45 s in a Gene Amp PCR system 2400 (Applied Biosystem, CA). PCR products were separated and electrophoresed on a 1.8% agarose gel and stained with Du Red (Biosharp, USA). They were then viewed under UV transillumination, and sizes were determined by comparison with a 50 bp DNA sequencing ladder. The* MAOA*-VNTR (MAOA promoter region polymorphism) was genotyped according to previously described methods [2]. Alleles with 2, 3, or 5 repeats were categorized as “low” activity, while those with 3.5 or 4 repeats were categorized as “high” activity.

### 2.4. Data Acquisition

All fMRI scans were obtained with a Philips Achieva 3-T scanner. The participants were instructed to keep their eyes closed and refrain from initiating goal-directed, attention-demanding activity during scanning. Foam pads were used to reduce head movements, and fitted ear plugs were used to reduce scanner noise. Resting-state fMRI scans were performed with an echo planar imaging sequence. Scan parameters were as follows: repetition time = 2000 ms; echo time = 30 ms; flip angle = 90°, matrix = 64 × 64; field of view = 240 mm; slice thickness = 4 mm; and slice gap = 0 mm. Each brain volume contained 36 axial slices, and each functional run contained 206 volumes. Before the functional scan, high-resolution (1 × 1 × 1 mm^3^) anatomical images of 180 contiguous slices were obtained with a 3D MPRAGE sequence (repetition time = 8.5 ms, echo time = 3.7 ms, and flip angle = 8°).

### 2.5. Data Preprocessing

Image preprocessing and statistical analyses were performed with Statistical Parametric Mapping (SPM8, http://www.fil.ion.ucl.ac.uk/spm/). The first six volumes of the functional images were discarded for magnetization equilibrium and participants' adaptation to scanning noise. For each participant, functional images were motion corrected using the realignment function. Data from the subjects whose head motions exceeded 2 mm in the *x*, *y*, or *z* plane or whose rotation exceeded 2° during scanning were excluded.

Each subject's anatomical images were segmented using the new segment function, and deformation field maps were obtained. The deformation field maps were applied to all functional images to normalize them into the standard Montreal Neurological Institute (MNI) space with a resampling voxel size of 3 × 3 × 3 mm^3^. Segmented anatomical images (unmodulated) were used to define white matter (WM) and cerebrospinal fluid (CSF) masks by thresholding the density images at a value of 0.99. For the time series of each voxel, the first eigenvector of the time series in the WM mask and the first eigenvector of the times series in the CSF mask, together with 24 motion parameters of Friston's model [38], were regressed out using linear regression. Finally, the time series of each voxel was filtered temporally using a band-pass filter (0.01–0.1 Hz) to reduce the effects of low-frequency drifts and of physiological high frequency respiratory and cardiac noises.

### 2.6. ALFF Analysis

The REST software package (REST, http://resting-fmri.sourceforge.net) was used to calculate ALFF values with a voxel-based approach. The filtered time series for each voxel were transformed to the frequency domain with a Fast Fourier Transform function, yielding a power spectrum. The square root of the power spectrum was calculated and then averaged across 0.01–0.08 Hz at each voxel. This averaged square root was taken as the ALFF [25]. To reduce global effects of variability across participants, the ALFF of each voxel was divided by the global mean ALFF value within the whole-brain mask obtained previously, and a standardized ALFF map of the whole brain was obtained. The ALFF maps were then smoothed spatially with an 8 mm, full width at half maximum (FWHM) Gaussian filter.

### 2.7. Analyses of Clinical Variables in relation to ALFF Data

To investigate the relationship between altered ALFF and impulsivity scores, the average ALFF values of all voxels were extracted separately, and then Pearson's correlation coefficients for ALFF values versus BIS-11 total scores were computed for each group separately.

### 2.8. Statistical Analysis

Two-sample *t*-tests were applied to assess demographic and clinical data differences between MAOA genotype groups in SPSS 11.6 software (SPSS Inc., Chicago, IL). To investigate ALFF differences between the two groups, two-sample *t*-tests were performed on the individual normalized ALFF maps in SPM8 software with age and educational level as nuisance covariates. The resulting statistical map was set at a combined threshold of *P* < .001 and a minimum cluster size of 50, which resulted in a cluster-corrected family-wise error *P* < .05.

## 3. Results

### 3.1. Genotyping and Participant Exclusions

The original cohort of 60 boys included 34 with the low-activity allele (57%) and 26 with the high-activity allele (43%). One high-activity allele and three low-activity allele subjects were excluded due to excessive head motion or rotation during scanning, leaving 31 low-activity allele and 25 high-activity allele subjects in the final analysis.

### 3.2. Demographic and Clinical Comparisons

The demographic and clinical data obtained for the two* MAOA* genotype groups are summarized in Table 1. There were no differences between the two genotype groups in terms of age, years of education, or BIS scores (all *P* > 0.05).

### 3.3. ALFF

**Figure 1:**
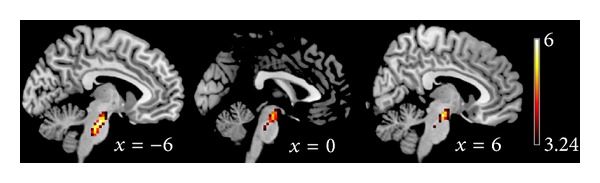
ALFF values using two-sample *t*-tests during resting state. Regions showing decreased ALFF values in male adolescents with MAOA-L compared to adolescents with MAOA-H were at the threshold *t* > 3.24, with correction for multiple comparisons applied at *P* < .05 (cluster-corrected with family wise error). Color bar indicates the *T* score.

Compared with MAOA-H carriers, adolescents carrying the MAOA-L allele showed significantly reduced ALFF values in the pons region of the brainstem (Table 2, Figure 1).

### 3.4. Behavioral Correlation

**Figure 2:**
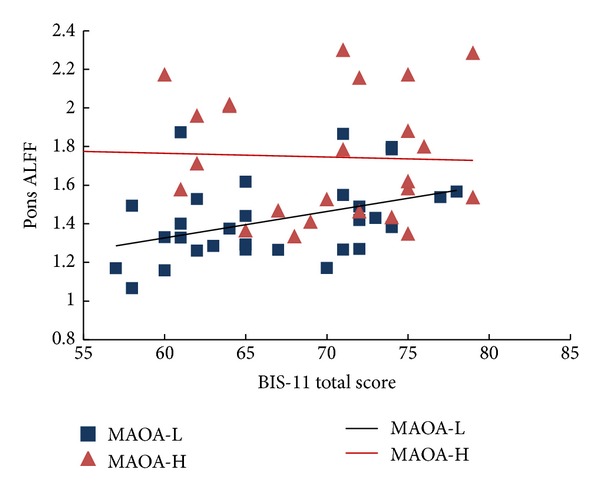
Correlations between BIS-11 total scores and pons ALFF values in MAOA-L and MAOA-H genotype groups.

Although there were no differences in BIS scores between the* MAOA* genotype groups, only the MAOA-L group showed a significant correlation between the BIS score and the ALFF values in the pons (*r* = 0.398, *P* = .02, Figure 2), MAOA-H group showed no such correlation (*r* = −0.044, *P* = .833, Figure 2). Further regression analysis showed a significant genotype by ALFF values interaction effect on BIS scores (*β* = 0.803, *P* < .05).

## 4. Discussion

To our knowledge, the present study is the first to reveal the influence of MAOA genotype on brain regional spontaneous activity in male adolescents. We found that male adolescents with the MAOA-L genotype had significantly lower ALFF values in the pons than MAOA-H counterparts. Given that ALFF changes are suggestive of regional spontaneous neuronal activity [25], this difference is consistent with the possibility that boys carrying the MAOA-L may have a neural impairment in the pons and points to differential effects on spontaneous brain activity between the two* MAOA* genotypes. The allelic distribution observed in our sample population was similar to those reported previously in Asian populations [39, 40], and the approximate inverse of ratios reported for Caucasian populations (60–70% high activity allele and 30–40% low activity allele) [5, 22].

BIS-11 scores did not differ significantly between the groups, likely due to the small sample size in our study. Interestingly, however, we found that ALFF values in the pons correlated with BIS scores selectively in boys with a MAOA-L genotype but not in MAOA-H group, and a significant genotype by ALFF interactive effect on impulsiveness. These results suggest that* MAOA* genotype may modulate the association of impulsiveness and ALFF in pons, and the association was only shown in MAOA-L group.

The pons, a major component of the brainstem, serves mainly as a relay center between the spinal cord, cerebellum, and cerebral cortices and neural centers that control respiration, heartbeat, reflexes, sleep, equilibrium, and auditory and visual functions [41, 42]. Within the pons region, the locus coeruleus and raphe nuclei are important cranial nerve nuclei and are the principal sites for brain synthesis of NE and 5-HT, respectively. Using* MAOA* cDNA and oligonucleotide probes, Jahng et al. found that the pons (especially the locus coeruleus) is the region with the highest density of* MAOA* mRNA in the rat brain [43]. The* MAOA* gene polymorphism regulates the metabolism of NE and 5-HT [1]; animal and human studies indicate that the serotonin system has an important effect on aggressive and impulsive behavior [44, 45]. Therefore, it is possible that* MAOA* genotype-dependent modulation of 5-HT and NE neurotransmission underlie the* MAOA* gene effect to impulsiveness. Besides, it is possible that the influence of* MAOA* on spontaneous neuronal activity in pons is most likely mediated through its effects on serotonin and dopamine.

Previous research has implicated the role of pons in aggression. For example, pontine lesions have been associated with aggressive behaviors in animals [39, 40]. A human PET study showed reduced brain MAOA activity in the pons of participants who had high trait aggression compared with that in nonaggressive participants [41]. Indeed, several previous reports have pointed to the MAOA-L allele as a “risk” variant (allele) for impulsivity and aggression. Since Caspi and colleagues discovered that males carrying MAOA-L alleles who experienced early-life adversity were significantly likely to evince conduct disorder, an antisocial personality, and exhibit violent, antisocial behavior [5], a number of investigators have replicated the similar founding [9–11]. In addition, Huang et al. found that people with MAOA-L allele displayed more aggression and impulsive tendencies experienced abuse relative to their peers with MAOA-H allele [7]. Our findings of low ALFF values in the pons of MAOA-L allele carriers, relative to those of MAOA-H allele carriers and selective correlation of BIS-11 scores with pons ALFF values in the MAOA-L group fit well the possibility that the MAOA-L allele may indeed be such a risk variant.

Our findings contrast with the results from a recent PET study showing no significant differences in resting brain glucose metabolism between the MAOA-L and MAOA-H genotypes [23]. However, previous fMRI studies have revealed significant effects of* MAOA* polymorphisms on brain activation in multiple corticolimbic regions, including anterior cingular cortex, orbitofrontal cortex, and amygdala [17–20]. The inconsistent findings might be due to the different methodologies used and differences in the participants' characteristics, especially with respect to age composition.

There are some limitations of the current study that warrant consideration. First, the sample size is really small. We cannot rule out the possibility of false positive findings and the possibility of the gene effect on other brain regions. Second, only males were included in our study. We cannot determine whether the results are specific to males or also relate to females. Future studies should include both males and females with a larger sample size. In addition, the* MAOA* promoter polymorphism is likely not the only* MAOA* variant that has an effect on impulsiveness or brain function. We cannot rule out the potential interactive contribution of genotype-phenotype and gene-environment to impulsiveness or brain function.

## 5. Conclusions

In summary, in the present study, we obtained evidence showing that a well-characterized functional polymorphism in the* MAOA* gene modulates resting-state spontaneous brain activity in the pons in healthy male adolescents. Lower∖spontaneous brain activity in the pons of the MAOA-L male adolescents may provide a neural mechanism by which boys with the MAOA-L genotype confers risk for impulsivity and aggression.

## Figures and Tables

**Table 1:** Demographic information of subjects.

Characteristic	MAOA-H (*N* = 25)	MAOA-L (*N* = 31)	*P* value
Age, years: mean (s.d.)	15.92 (0.81)	15.55 (0.81)	0.09
Education, year: mean (s.d.)	10.56 (0.51)	10.52 (0.57)	0.77
BIS-11 score: mean (s.d.)	69.16 (7.17)	66.71 (6.05)	0.17

Abbreviations: MAOA-H = high-activity MAOA genotype group; MAOA-L = low-activity MAOA genotype group;

BIS-11 = Italian version of the 11-item Barratt's Impulsivity Scale; s.d. = standard deviation.

**Table 2:** Regions that showed significant differences in ALFF values between the high-activity and low-activity MAOA genotype groups in resting-state.

Bran region	Side	Peak MNI coordinates (mm)			Cluster size	*Z* score	*P* value*
		*X*	*Y*	*Z*			
MAOA-L < MAOA-H							
Pons	L	−6	−19	−23	130	4.38	<0.001
	R	6	−16	−17		4.12	
	L	−6	−25	−32		4.01	

Abbreviations: ALFF= amplitude of low-frequency fluctuations; MAOA-H = high-activity MAOA genotype group; MAOA-L = low-activity MAOA genotype group; L= left; R= right; MNI= Montreal Neurological Institute.

*Cluster-corrected with family-wise errors.
